# *Staphylococcus aureus* convert neonatal conventional CD4^+^ T cells into FOXP3^+^ CD25^+^ CD127^low^ T cells via the PD-1/PD-L1 axis

**DOI:** 10.1111/imm.12209

**Published:** 2014-02-10

**Authors:** Hardis Rabe, Inger Nordström, Kerstin Andersson, Anna-Carin Lundell, Anna Rudin

**Affiliations:** Department of Rheumatology and Inflammation Research, The Sahlgrenska Academy at University of GothenburgGothenburg, Sweden

**Keywords:** cord blood, FOXP3^+^ regulatory T cells, Helios, programmed cell death 1, *Staphylococcus aureus*

## Abstract

The gut microbiota provides an important stimulus for the induction of regulatory T (Treg) cells in mice, whether this applies to newborn children is unknown. In Swedish children, *Staphylococcus aureus* has become a common early colonizer of the gut. Here, we sought to study the effects of bacterial stimulation on neonatal CD4^+^ T cells for the induction of CD25^+^ CD127^low^ Treg cells *in vitro*. The proportion of circulating CD25^+^ CD127^low^ Treg cells and their expression of FOXP3, Helios and CTLA-4 was examined in newborns and adults. To evaluate if commensal gut bacteria could induce Treg cells, CellTrace violet-stained non-Treg cells from cord or peripheral blood from adults were co-cultured with autologous CD25^+^ CD127^low^ Treg cells and remaining mononuclear cells and stimulated with *S. aureus*. Newborns had a significantly lower proportion of CD25^+^ CD127^low^ Treg cells than adults, but these cells were Helios^+^ and CTLA-4^+^ to a higher extent than in adults. FOXP3^+^ CD25^+^ CD127^low^ T cells were induced mainly in neonatal CellTrace-stained non-Treg cells after stimulation with *S. aureus*. In cell cultures from adults, *S. aureus* induced CD25^+^ CD127^low^ T cells only if sorted naive CD45RA^+^ non-Treg cells were used, but these cells expressed less FOXP3 than those induced from newborns. Sorted neonatal CD25^+^ CD127^low^ T cells from *S. aureus*-stimulated cultures were still suppressive. Finally, blocking PD-L1 during stimulation reduced the induction of FOXP3^+^ CD25^+^ CD127^low^ T cells. These results suggest that newborns have a higher proportion of circulating thymically derived Helios^+^ Treg cells than adults and that *S. aureus* possess an ability to convert neonatal conventional CD4^+^ T cells into FOXP3^+^ CD25^+^ CD127^low^ Treg cells via the PD-1/PD-L1 axis.

## Introduction

Regulatory T (Treg) cells are essential for the maintenance of self tolerance and immune homeostasis. Mice that lack Treg cells suffer from severe autoimmune and inflammatory conditions,^1^ and children with a FOXP3 gene mutation develop autoimmune diseases, severe dermatitis and food allergy with enterocolitis.^2^,^3^ Human Treg cells suppress proliferation and cytokine production of other T cells in response to self or environmental antigens.^4^,^5^ The Treg cells are found within the CD4^+^ CD25^+^ T-cell population and they express the transcription factor forkhead box P3 (FOXP3)^6^ and cytotoxic T lymphocyte antigen 4 (CTLA-4),^7^ the latter a molecule proven essential for its regulatory function.^8^ Moreover, Treg cells express little or no CD127 on the cell surface.^9^ Indeed, CD127 expression correlates inversely with FOXP3 expression and inhibitory function of human CD4^+^ Treg cells.^9^ It is still unknown if there is a difference in the intracellular expression of FOXP3 or CTLA-4 in CD4^+^ CD25^+^ CD127^low^ Treg cells from newborn children and adults, although Treg cells from newborns and adults have been shown to suppress equally well.^9^

Treg cells can be divided into two major subsets: thymically derived Treg (tTreg) cells and peripherally derived Treg (pTreg) cells generated in the periphery from naive CD4^+^ FOXP3^neg^ T cells.^10^ It is difficult to discriminate between tTreg and pTreg cells because both share similar molecular signatures, including high expression of surface CD25 and intracellular FOXP3 and CTLA-4, but no or low expression of surface CD127. Helios, an Ikaros family transcription factor, is up-regulated in FOXP3^+^ Treg cells, and it has been suggested that it would be selectively expressed in tTreg cells as opposed to in pTreg cells.^11^ However, this notion has been challenged as Helios expression is induced upon activation in CD4^+^ T cells, CD8^+^ T cells and Treg cells,^12^ and a population of Helios^neg^ CD45RA^+^ CD62L^+^ CCR7^+^ CD31^+^ tTreg cells was recently discovered in adults.^13^ It is not known whether CD25^+^ CD127^low^ Treg cells in newborn children and adults differ in Helios expression and we hypothesized that newborn children would express a higher proportion of Helios^+^ CD25^+^ CD127^low^ Treg cells than adults.

Next to tTreg cells, induction of pTreg cells is of importance for immune homeostasis and might be a target for the prevention of allergic and autoimmune disorders. Suppressive CD25^+^ FOXP3^+^ Treg cells can be induced from naive CD4^+^ FOXP3^neg^ T cells *in vitro*^14^ and *in vivo*,^15^ but the molecular mechanisms involved in the induction of Treg cells are not fully understood. Some reports suggest that efficient induction of FOXP3^+^ cells occur during conditions that are suboptimal for general T-cell activation, i.e. stimulation with low antigen concentrations and lack of co-stimulation by antigen-presenting cells (APCs), arguing that weak T-cell receptor stimulation favours the generation of pTreg cells.^16^,^17^ Furthermore, the expression of the inhibitory molecule programmed cell death ligand 1 (PD-L1) on APCs has been linked with their ability to induce FOXP3^+^ Treg cells.^18^–^20^ Interaction between PD-L1 and its receptor programmed cell death 1 (PD-1) expressed on activated T cells impede the T-cell receptor signalling pathway within the T cell,^18^,^21^ which can lead to differentiation into FOXP3^+^ Treg cells.^18^,^19^,^22^

Experiments in germ-free mice have shown that bacterial colonization of the intestine is of great importance for the induction of Treg cells,^23^–^26^ as these mice have a lower proportion of and less suppressive FOXP3^+^ Treg cells in the gut compared with conventional mice.^25^ When germ-free mice are colonized with either a mixture of different bacterial strains, a mixture of *Clostridium* species or with *Bacteroides fragilis* the proportion of FOXP3^+^ Treg cells in the gut is increased.^23^–^26^ These experiments also show that bacterial stimulation induces *de novo* generation of FOXP3^+^ pTreg cells because higher proportions of the Helios^neg^ Treg cells were found in the gut of colonized mice compared with in germ-free mice.^25^,^26^

In developing countries, the first bacteria that colonize the infantile gut include *Escherichia coli*, enterobacteria (other than *E. coli*) and enterococci.^27^ In Swedish infants, however, colonization with *E. coli* is delayed and coagulase-negative staphylococci and/or *Staphylococcus aureus* are the first colonizers, possibly due to reduced competition from traditional faecal bacteria.^28^ Whether or not certain commensal bacteria from the gut can induce Treg cells in newborn infants is unknown. However, we have shown that infants who harboured *S. aureus* in the gut during the first week(s) of life had a decreased risk of developing food allergy compared with children devoid of this bacterium.^29^ Further, infants who received oral supplementation with *Lactobacillus reuteri* during the first year of life have lower prevalence of IgE-mediated eczema at 2 years of age than the placebo group.^30^ Moreover, certain lactobacilli species have been shown to induce FOXP3^+^ Treg cells *in vitro* using cells from adult individuals.^31^

The present study demonstrates that a higher proportion of Treg cells from newborn children are naive and express Helios and CTLA-4 relative to adults. Furthermore, cell tracking of neonatal CD4^+^ non-Treg cells stimulated with *S. aureus* revealed *de novo* generation of FOXP3^+^ CD25^+^ CD127^low^ cells. The CD25^+^ CD127^low^ T cells from *S*. *aureus*-stimulated cultures were still functional as they efficiently suppressed CD4^+^ T-cell proliferation. Stimulation with *S*. *aureus* also increased the proportion of B cells that express PD-L1 compared with unstimulated control cultures, in cultures from both cord and peripheral blood from adults. Blocking PD-L1 during stimulation with *S. aureus* repressed the induction of neonatal FOXP3^+^ CD25^+^ CD127^low^ T cells. Taken together, these results suggest that *S. aureus* possess the ability to induce T-cell populations with immunoregulatory functions early in infancy.

## Materials and methods

### Subjects and collection of blood samples

Cord blood samples were collected from unselected healthy newborn infants born at term (≥ 38 weeks of gestation) at the Sahlgrenska University Hospital and peripheral blood was obtained from healthy adult volunteers with no relation to the newborn children. All parents and adult volunteers were given oral and written information, and gave oral consent to participate in the study. Ethical approval was obtained through the Human Research Ethics Committee of the Medical Faculty, University of Gothenburg, Sweden.

### Bacterial strains

Bacterial strains from the commensal intestinal flora of healthy Swedish infants, including *S. aureus* and *Lactobacillus paracasei* were isolated from stool samples as previously described in detail.^28^ Before use in cell culture, all bacterial strains were counted in a microscope and killed by exposure to UV light for 20–30 min, which was confirmed by negative viable count. Bacteria were then stored at −70° until use.

### Flow cytometry

**Figure 1:**
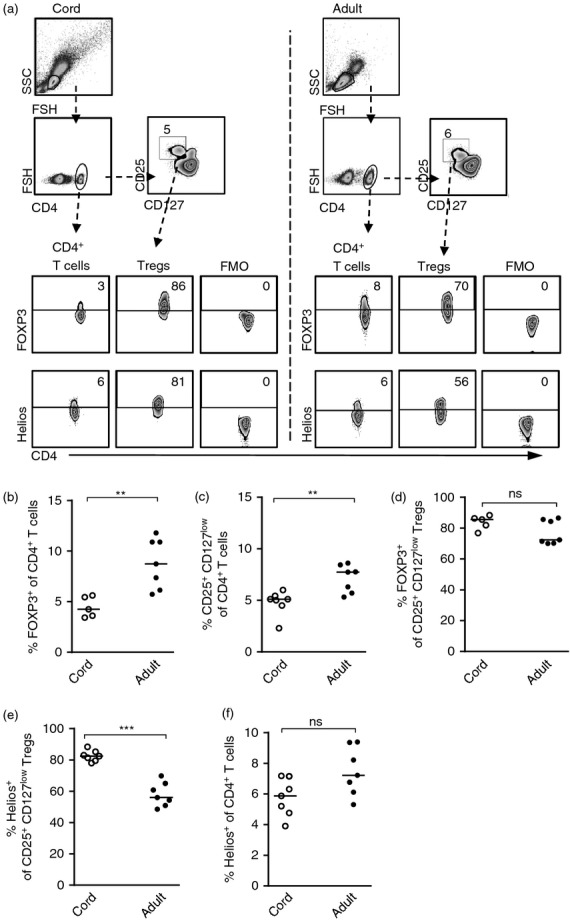
The proportion of Helios expressing regulatory T (Treg) cells and CD4^+^ T cells in newborn infants and in adults. (a) Gating strategies for FOXP3 and Helios expression, as well as the fluorochrome minus one control, within the CD4^+^ T cells and CD4^+^ CD25^+^ CD127^low^ Treg cells in newborns and in adults. Numbers represent the percentage of cells within the gate and for each sample 10 000–20 000 CD4^+^ T cells were collected. (b) The proportion of CD4^+^ FOXP3^+^ T cells in cord or peripheral blood from adults. (c) The proportion of CD25^+^ CD127^low^ Treg cells of CD4^+^ T cells in blood from newborns and adults. (d–f) The percentage of (d and e) CD25^+^ CD127^low^ Treg cells or (f) CD4^+^ T cells that express (d) FOXP3 or (e and f) Helios in newborns and in adults. Each symbol represents one individual and horizontal bars indicate the median value. ** *P* ≤ 0·01 and *** *P* ≤ 0·001 (two-tailed Mann–Whitney *U*-test).

**Figure 2:**
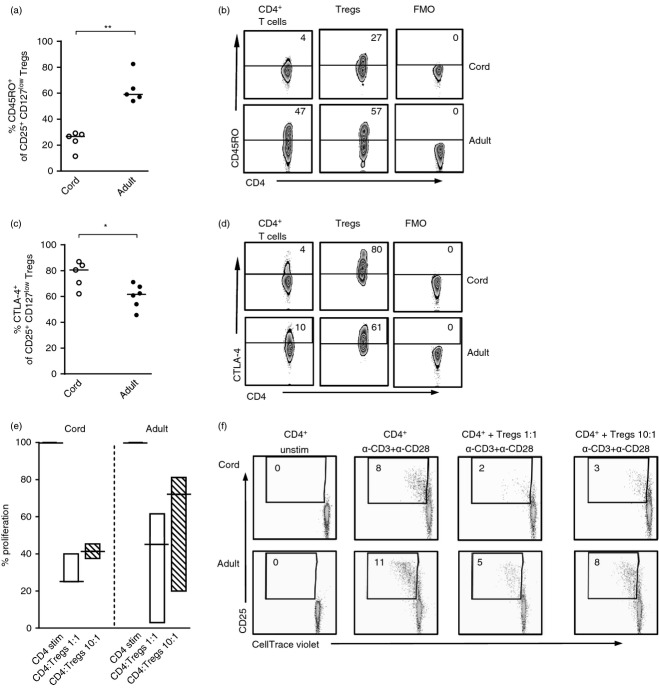
The proportion of CD25^+^ CD127^low^ regulatory T (Treg) cells that express CD45RO or CTLA-4 in newborn infants and in adults. The proportion of CD4^+^ CD25^+^ CD127^low^ Treg cells in cord blood and peripheral blood from adults that express (a and b) CD45RO or (c and d) CTLA-4. Each symbol represents one individual and horizontal bars indicate the median value. Representative dot plots showing the proportion of CD4^+^ T cells and Treg cells that express (b) CD45RO or (d) CTLA-4 as well as the fluorochrome minus one control in newborn children and adults. (e and f) CellTrace violet stained CD4^+^ CD25^neg^ responder T cells were stimulated with *α*-CD3 and *α*-CD28 and cultured alone or in the presence of sorted autologous CD25^+^ CD127^low^ Treg cells in the ratios 1 : 1 or 10 : 1, illustrated in (e) a graph that shows the median percentage with minimum to maximum value of proliferated CD4^+^ CD25^neg^ responder cells in the presence of Treg cells (ratio: 1 : 1 white bars or 10 : 1 striped bars) compared with cultures stimulated without Treg cells (black line) and (f) representative dot plots. The graph and dot plots show data from three experiments performed on blood from three different infants and adults. Numbers within the FACS plots represent the percentage of cells within the gate and for each sample 10 000–20 000 CD4^+^ T cells were collected. *P ≤ 0·05, **P ≤ 0·01 (two-tailed Mann–Whitney *U*-test).

**Figure 3:**
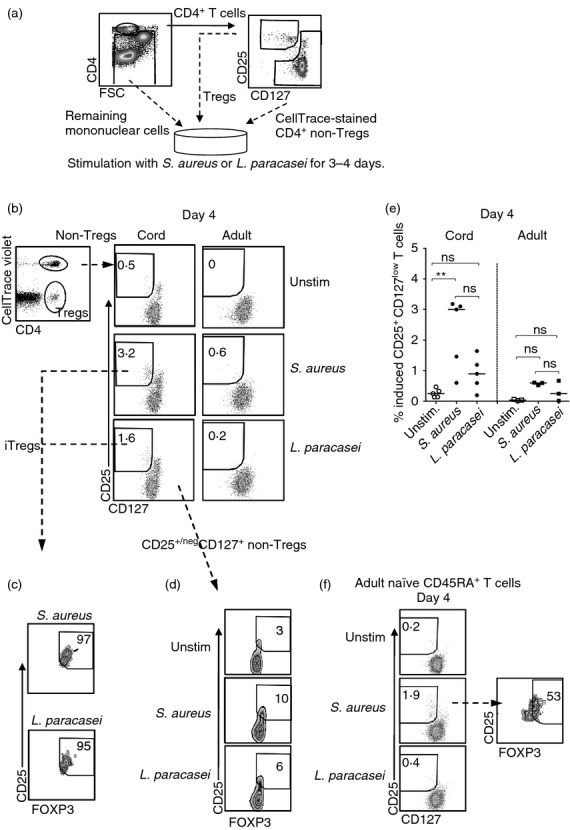
Stimulation with *Staphylococcus aureus* induces CD25^+^ CD127^low^ T cells from non-regulatory T (non-Treg) cells. (a) CD4^+^ CD25^neg/+^ CD127^+^ (non-Treg cells), CD4^+^ CD25^+^ CD127^low^ T cells (Treg cells) and remaining mononuclear cells from cord or adult peripheral blood were sorted using flow cytometry. Next, non-Treg cells were stained with CellTrace violet before they were co-cultured with Treg cells and the remaining mononuclear cells in the presence of *S. aureus* or *Lactobacillus paracasei* for 3–4 days. (b) Representative dot plots of the proportion of CD25^+^ CD127^low^ T cells within the CellTrace^+^ CD4^+^ non-Treg cell population after bacterial stimulation for 4 days, in cell cultures from newborns (*n* = 5) and adults (*n* = 3). (c and d) Zebra plots illustrate the proportion of FOXP3^+^ cells within the (c) induced Treg cell population or (d) the CD25^neg/+^ CD127^+^ non-Treg cells from the experiment shown in (b). (e) The proportion of *de novo*-generated CD25^+^ CD127^low^ T cells within the CD4^+^ non-Treg cell population after bacterial stimulation for 4 days in cultures from newborns and adults. Each symbol in the graph represents one individual and horizontal bars indicate the median value. (f) Representative dot plots of the proportion of adult CD25^+^ CD127^low^ T cells within the CellTrace^+^ CD4^+^ non-Treg cell population after bacterial stimulation of CD45RA^+^ CD4^+^ non-Treg cells, autologous Treg cells and remaining mononuclear cells for 4 days. The dot plots represent one experiment of two performed on peripheral blood from two adult individuals. For each sample 3000–20 000 CellTrace^+^ CD4^+^ T cells were collected. ** *P* ≤ 0·01 (Kruskal–Wallis test followed by Dunn's multiple comparison test).

**Figure 4:**
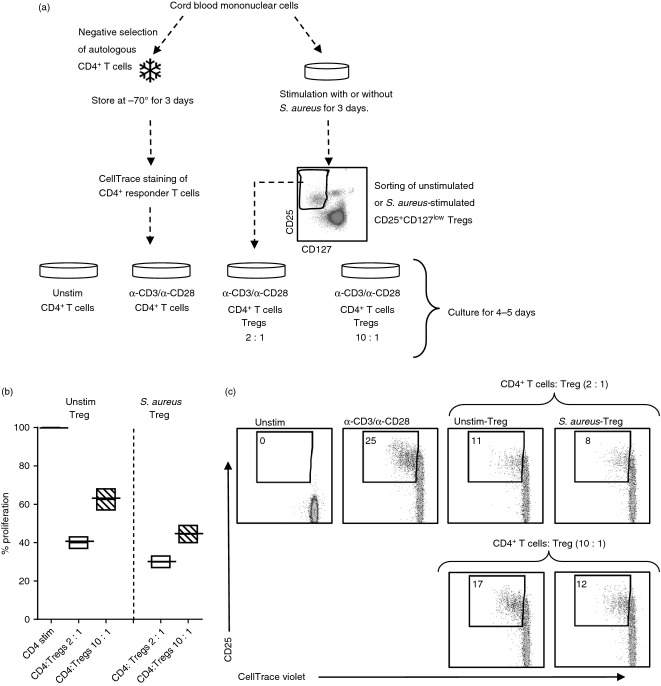
CD25^+^ CD127^low^ T cells from cultures stimulated with *Staphylococcus aureus* suppress proliferation of CD4^+^ responder cells. (a) CD4^+^ CD25^neg^ responder T cells were isolated and stored for 3 days at −70°. On day 3 the responder cells were thawed and stained with CellTrace violet. Thereafter, the stained responder cells were stimulated with *α*-CD3 and *α*-CD28 and co-cultured in a ratio of 2 : 1 or 10 : 1 for 3–5 days with or without sorted autologous regulatory T (Treg) cells that had been cultured in the absence or in the presence of *S. aureus*. (b) The median percentage with minimum to maximum values of proliferated CD4^+^ CD25^neg^ responder cells in the presence of unstimulated or *S. aureus* stimulated Treg cells (ratio 2 : 1 white bars, ratio 10 : 1 striped bars) compared with cultures stimulated without Treg cells (black line). (c) Representative dot plots on the CD4^+^ responder cells stimulated with *α*-CD3 and *α*-CD28 alone or co-cultured with unstimulated Treg cells or *S. aureus*-induced Treg cells in a ratio of 2 : 1 or 10 : 1. Numbers represent the percentage of cells within the gate for dividing cells. For each sample, 10 000 CD4^+^ T cells were collected and the dot plots represent one experiment of three performed on cord blood from different infants.

**Figure 5:**
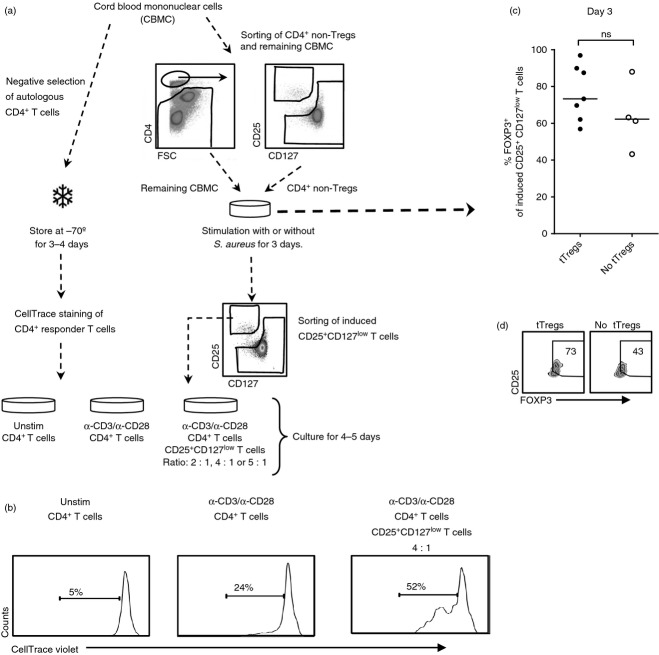
Stimulation with *Staphylococcus aureus* in the absence of pre-existing regulatory T (Treg) cells induces activated FOXP3^low^ CD25^+^ CD127^low^ T cells. (a) CD4^+^ CD25^neg^ responder T cells were isolated and stored for 3 days at −70°. CD4^+^ CD25^neg/+^ CD127^+^ (non-Treg cells), CD4^+^ CD25^+^ CD127^low^ T cells (Tregs) and remaining mononuclear cells from the same cord blood sample were sorted using flow cytometry. Next, non-Treg cells and the remaining mononuclear cells were co-cultured and stimulated with *S. aureus* for 3 days. On day 3 the responder cells were thawed and stained with CellTrace violet, stimulated with *α*-CD3 and *α*-CD28 and co-cultured in a ratio of 2 : 1, 4 : 1 or 5 : 1 (depending on the quantity of CD25^+^ CD127^low^ T cells that was collected during sorting), for 4–5 days with sorted autologous *S. aureus*-induced CD25^+^ CD127^low^ T cells (*n* = 3). (b) Representative histogram on the proliferation of CD4^+^ responder cells stimulated with *α*-CD3 and *α*-CD28 alone or co-cultured with induced CD25^+^ CD127^low^ T cells after stimulation with *S. aureus* in the absence of pre-existing regulatory T (Treg) cells (tTregs) during the stimulation phase in a ratio of 4 : 1. (c and d) The proportion of induced CD25^+^ CD127^low^ T cells that express FOXP3 in cultures in which non-Treg cells and remaining cord blood mononuclear cells were stimulated with *S. aureus* in the presence (*n* = 7) or absence (*n* = 4) of tTreg cells for 3 days, illustrated in (c) a graph and (d) zebra plots of one individual. The numbers represent the percentage of cells within the gate and for each sample, 8000–10 000 CD4^+^ T cells were collected. Each symbol in the graphs represents one individual and horizontal bars indicate the median value.

**Figure 6:**
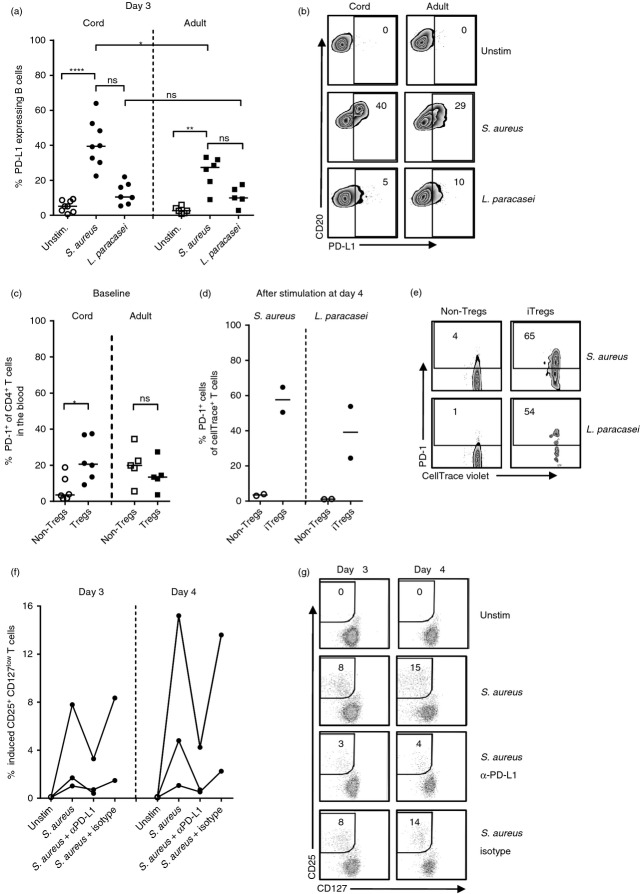
Blocking programmed death 1 (PD-1)/ programmed death ligand 1 (PD-L1) interaction during *Staphylococcus aureus* stimulation reduces induction of neonatal FOXP3^+^ CD25^+^ CD127^low^ T cells. (a and b) The proportion of PD-L1^+^ B cells after stimulation of mononuclear cells from cord blood and peripheral blood from adults with *S. aureus* or *Lactobacillus paracasei* for 3 days, illustrated in (a) a graph and (b) representative zebra plots. (c–e) The expression of PD-1 on (c) circulating non-regulatory T (non-Treg) cells and CD25^+^ CD127^low^ T cells in newborns and adults and (d and e) *S. aureus*-stimulated non-Treg cells and *S. aureus*-induced Treg cells after 4 days of culture, illustrated in (c and d) graphs and (e) representative zebra plots. (f and g) The proportion of *de novo* generated CD25^+^ CD127^low^ T cells within the CD4^+^ non-Treg cell population after stimulation with *S. aureus* in the presence or absence of *α*-PD-L1 (*n* = 3) or isotype control (*n* = 2) for 3–4 days, illustrated in (f) a graph and (g) representative zebra plots. Each symbol in the graphs represents one individual and horizontal bars indicate the median value. Numbers represent the percentage of cells within the gate. (Kruskal–Wallis test followed by Dunn's multiple comparison test was used to compare the proportions of PD-L1^+^ B cells among unstimulated control and stimulated cultures, Mann–Whitney *U*-test was used to compare the proportion of PD-L1^+^ B cells among cultures from cord and adult blood after *S. aureus* or *L. paracasei* stimulation, and Wilcoxon signed rank test was used to compare the expression of PD-1 on non-Treg cells and Treg cells). * *P* ≤ 0·05, ** *P* ≤ 0·01, ****P ≤ 0·0001.

Flow cytometric analysis was either performed on freshly separated mononuclear cells from cord and adult blood, isolated by density gradient centrifugation (900 ***g***, 20 min at room temperature) over Lymphoprep™ (Nycomed, Oslo, Norway), or on cells cultured for 3–4 days in the presence or absence of commensal bacteria as described below. For cell surface staining, the cells were incubated with monoclonal antibodies for 20 min at 4° in the dark. Thereafter, the cells were washed twice and suspended in FACS buffer before analysis. For intracellular markers, surface stained cells were fixed, permeabilized and incubated with monoclonal antibodies against FOXP3, Helios or CTLA-4. The following anti-human monoclonal antibodies were used: Figs. 1 and 2, V450 or allophycocyanin-H7-conjugated anti-CD4 (V450-clone RPA-T4 and allophycocyanin-H7-clone SK3), FITC- or Alexa Fluor 647-conjugated anti-CD127 (clone HIL-7R-M21), phycoerythrin- (PE), PE-Cy7- or Brilliant violet 421-conjugated anti-CD25 (PE and PE-cy7-clone 2A3 and Brilliant violet 421-clone BC96) and PE-conjugated anti-CD45RO (clone UCHL1); Figs. 3–6, PE-, allophycocyanin- or Brilliant violet-conjugated CD25 (PE and allophycocyanin clone 2A3 and Brilliant violet 421-CD25 clone BC96), FITC- or Alexa Fluor 647-conjugated CD127 (clone HIL-7R-M21), allophycocyanin-H7-conjugated CD20 (clone L27), Brilliant violet 421-conjugated PD-L1 (clone 29E.2A3), and allophycocyanin-H7-conjugated anti-CD4 (clone SK3). All antibodies were purchased from BD Bioscience (San Jose, CA) except for Brilliant violet-conjugated CD25 and PD-L1, which were purchased from BioLegend (San Diego, CA). Fixation and permeabilization were performed using the kit Human FOXP3 Buffer set (BD Bioscience), followed by intracellular staining with PE-conjugated anti-FOXP3 (clone PCH101, BD Bioscience), Alexa Fluor 488-conjugated anti-Helios (clone 22F6, BioLegend) or biotin-conjugated CTLA-4 (clone BNI3, BD Bioscience), followed by PE-conjugated streptavidin (BD Bioscience). The samples were analysed on a FACSCanto II (BD Biosciences), equipped with FACSDiva software. All flow cytometry data was analysed using flowjo software (Tree Star, Ashland, OR). The fluorochrome minus one method was used for gating strategy, as described previously.^32^

### Cell culture

To examine if Treg cells could be induced from non-Treg cells, mononuclear cells from cord (CBMC) or adult peripheral blood (PBMC) were isolated by density gradient centrifugation (900 ***g***, 20 min at room temperature) over Lymphoprep™ and stained with FITC-conjugated anti-CD4, Alexa Fluor 647-conjugated anti-CD127 (BD Bioscience), and Brilliant violet 421-conjugated CD25 (BioLegend) for 20 min at 4°. Thereafter, CD4^+^ CD25^+/neg^ CD127^+^ T cells (non-Treg cells), CD4^+^ CD25^+^ CD127^low^ (Treg cells) and remaining CBMC or PBMC were sorted using an iCyt Synergy™ cell sorter (Sony Biotech, Champaign, IL). The CD4^+^ CD25^+/neg^ CD127^+^ T cells (purity after sorting 98–99%) were stained with 4 μg/ml CellTrace Violet (Invitrogen, Eugene, OR) according to manufacturer's recommendations and co-cultured at a concentration of 40 × 10^3^ cells/well with autologous CD4^+^ CD25^+^ CD127^low^ Treg cells (2 × 10^3^ cells/well) (purity after sorting 97–99%) and the remaining CBMC or PBMC (158 × 10^3^/well). The cells were stimulated with 5 × 10^7^/ml of commensal *S. aureus* (a strain able to produce staphylococcal enterotoxin C before being UV-killed) or *L. paracasei*.

In some cultures (depicted in Fig. 3f), naive CD4^+^ CD45RA^+^ CD25^neg^CD127^+^ T cells (purity after sorting 97–98%) from adult blood were sorted and stained with CellTrace violet before stimulation with bacteria in the co-culture with autologous CD4^+^ CD25^+^ CD127^low^ Treg cells and remaining mononuclear cells. In the Supplementary material (Fig. S2), CD4^+^ CD25^+/neg^ CD127^+^ T cells and remaining CBMC were cultured without CD4^+^ CD25^+^ CD127^low^ Treg cells. When blocking PD-L1, the remaining CBMC were first stimulated with *S. aureus* in the presence or absence of *α*-PD-L1 (at a final concentration of 10 μg/ml in the culture, clone MIH1, eBioscience, San Diego, CA) or isotype control IgG1 (clone MG1-45, eBioscience) for 45 min before adding the autologous Treg cells and CellTrace violet-stained non-Treg cells (Fig. 6f,g). All cultures were performed in U-bottomed 96-well culture plates in RPMI-1640 media (PAA Laboratories GmbH, Pasching, Austria) supplemented with 5% heat-inactivated autologous plasma, 1 mm l-glutamine and 50 μg/ml gentamycin for 3–4 days, kept in 5% CO_2_ at 37°.

To investigate whether stimulation with *S. aureus* or *L. paracasei* would increase the proportion of B cells that expressed PD-L1, mononuclear cells were first isolated from cord blood or peripheral blood from adults as described above. Next, 1 × 10^6^ mononuclear cells/ml were cultured with or without 5 × 10^7^/ml of commensal *S. aureus* or *L. paracasei* in U-bottomed 96-well culture plates in supplemented RPMI-1640 media for 72 hr, kept in 5% CO_2_ at 37°.

### Suppression assay

To examine the suppressive capacity of freshly isolated CD4^+^ CD25^+^ CD127^low^ Treg cells, cord or adult blood mononuclear cells were stained with FITC-conjugated anti-CD4, Alexa Fluor 647-conjugated anti-CD127 and Brilliant violet 421-conjugated CD25 for 20 min at 4°. Thereafter, CD4^+^ CD25^+^ CD127^low^ Treg cells were sorted in an iCyt Synergy™ cell sorter (purity after sorting 93–97%). Autologous CD4^+^ CD25^neg^ responder cells were enriched from the same samples using a Miltenyi Biotec CD4^+^ T-cell Isolation Kit II (Miltenyi Biotec, Bergisch Gladbach, Germany), and stained with 4 μg/ml CellTrace Violet. Responder cells (8 × 10^4^ cells/well) were cultured in the presence or absence of plate-bound *α*-CD3 (1·5 μg/ml, clone OKT-3, Ortho-McNeil Pharmaceutical, Raritan, NJ) and soluble *α*-CD28 (1 μg/ml, clone CD28.2, BD BioScience) and with or without autologous Treg cells in different ratios (1 : 1 or 10 : 1). The ability of Treg cells to suppress CD4^+^ responder cells was examined after 4–5 days of co-culture. Cultured cells were washed and stained with FITC-conjugated anti-CD4 and allophycocyanin-conjugated anti-CD25 (BD Bioscience) to study the proliferation of the responder cells.

To study the suppressive capacity of both pre-existing and induced Treg cells derived from cultures stimulated with *S. aureus*, CD4^+^ responder T cells (CD4^+^ CD25^neg^) were isolated using Miltenyi's CD4^+^ T cell Isolation Kit II and thereafter gradually cooled and stored at −70° for 3 days. From the same cord blood sample, mononuclear cells were adjusted to 1 × 10^6^/ml and stimulated with 5 × 10^7^/ml commensal UV-killed *S. aureus* in flat-bottomed 24-well plates in supplemented RPMI-1640 media (as described above) for 3 days, kept in 5% CO_2_ at 37°. On day 3, CD4^+^ responder T cells were gradually thawed and stained with 4 μg/ml CellTrace violet. CD4^+^ CD25^+^ CD127^low^ Treg cells were sorted (purity after sorting 95–98%) from unstimulated or *S. aureus-*stimulated cultures and co-cultured in a ratio of 1 : 2 (40 × 10^3^ Treg cells : 80 × 10^3^ CD4^+^ responder cells) or 1 : 10 (8 × 10^3^ Treg cells : 80 × 10^3^ CD4^+^ cells) with autologous CellTrace violet-stained CD4^+^ responder cells in the presence of plate-bound *α*-CD3 and soluble *α*-CD28. The cells were cultured for 4–5 days in 5% CO_2_ at 37° in X-vivo 15 medium (Lonza, Verviers, Switzerland). To measure the proliferation of the responder cells, the cells were washed and stained with FITC-conjugated CD4 and APC-conjugated CD25.

To analyse whether *S. aureus*-induced CD25^+^ CD127^low^ T cells were suppressive, CD4^+^ responder T cells (CD4^+^ CD25^neg^) were isolated from CBMC as described above and stored at −70°. CD4^+^ CD25^+/neg^ CD127^+^ T cells (non-Treg cells), CD4^+^ CD25^+^ CD127^low^ (Treg cells) and remaining CBMC, from the same cord blood sample were sorted using an iCyt Synergy™ cell sorter (Sony Biotech). The pre-existing CD4^+^ CD25^+^ CD127^low^ Treg cells were discarded. Next, 20 × 10^4^ cells/well CD4^+^CD25^+/neg^CD127^+^ non-Treg cells (purity after sorting 98–99%) were co-cultured with autologous remaining CBMC (80 × 10^4^cells/well), and stimulated with or without 5 × 10^7^/ml of *S. aureus* in 24-well culture plates. On day 3–4, CellTrace violet^neg^ CD25^+^ CD127^low^ T cells were sorted (purity after sorting 95–99%) from *S. aureus-*stimulated cultures. The CD4^+^ responder T cells were thawed, stained with 4 μg/ml CellTrace violet and co-cultured at a ratio of 1 : 2 (10 × 10^3^ CD25^+^ CD127^low^ T cells : 20 × 10^3^ CD4^+^ responder cells), 1 : 4 (5 × 10^3^ CD25^+^ CD127^low^ T cells : 20 × 10^3^ CD4^+^ responder cells) or 1 : 5 (8 × 10^3^ CD25^+^ CD127^low^ T cells : 40 × 10^3^ CD4^+^ cells) with the sorted CD25^+^ CD127^low^ T cells, depending on how many CD25^+^ CD127^low^ T cells were collected during sorting. The cells were stimulated with plate-bound *α*-CD3 and soluble *α*-CD28 as described above. After 4–5 days of culture, the cells were washed and stained with FITC-conjugated CD4 and PE-conjugated CD25 and the proliferation of the responder cells was studied as described above.

### Statistical analysis

Mann–Whitney *U*-test, Kruskal–Wallis test followed by Dunn's multiple comparison test or Wilcoxon signed rank test (graphpad prism; GraphPad, San Diego, CA) were used as described in the figure legends. *P *≤* *0·05 was considered as significant (*P ≤* *0·05, **P ≤* *0·01, ***P ≤* *0·001 and ****P ≤* *0·0001).

## Results

### The proportion of Helios^+^ CD25^+^ CD127^low^ Treg cells is higher in newborn infants than in adults

To compare the proportion of Treg cells using two strategies of defining these cells, we first compared the proportion of CD4^+^ FOXP3^+^ with that of CD4^+^ CD25^+^ CD127^low^ Treg cells in newborn children and adults (gating strategy demonstrated in Fig. 1a). As demonstrated previously,^33^ we found that adults had a significantly higher proportion of circulating Treg cells than newborns with respect to both CD4^+^ FOXP3^+^ Treg cells (Fig. 1a,b) and CD4^+^ CD25^+^ CD127^low^ Treg cells (Fig 1a and c) and that the proportions of these two different Treg cell phenotypes were comparable. The majority of the CD25^+^ CD127^low^ Treg cells expressed FOXP3 in both neonates and adults (Fig. 1a and d). Furthermore, we found that newborn children had a higher fraction of Helios^+^ CD25^+^ CD127^low^ Treg cells compared with adults (Fig. 1a and e), whereas there was no difference in Helios expression within the total CD4^+^ T-cell population (Fig. 1a and f). These results indicate that there is a higher fraction of circulating tTreg cells in newborn children relative to adults.

The expression of CD45RO and CTLA-4 was also examined in the CD25^+^ CD127^low^ Treg cells. As expected, the proportion of CD4^+^ T cells and Treg cells that expressed CD45RO was significantly lower in newborns compared with adults (Fig. 2a,b). In contrast, newborn children had a higher proportion of CD25^+^ CD127^low^ Treg cells that expressed CTLA-4 than adults (Fig. 2c,d). Within the total CD4^+^ T-cell population, however, newborns had a lower proportion of CTLA-4 compared with adults (Fig. 2d, *P* = 0·001). Freshly isolated CD25^+^ CD127^low^ Treg cells from both cord and adult blood were able to suppress the proliferation of autologous responder cells when present in a 1 : 1 and 1 : 10 ratio (Fig. 2e,f). Hence, we conclude that cord blood CD25^+^ CD127^low^ Treg cells express higher amounts of CTLA-4 and are at least as suppressive as those of adults, despite the majority of the neonatal Treg cells being naive.

### *De novo* generation of neonatal FOXP3^+^ CD25^+^ CD127^low^ T cells after stimulation with *S. aureus*

To examine whether bacterial stimulation might induce *de novo* generation of CD25^+^ CD127^low^ T cells, sorted and CellTrace violet-stained non-Treg cells (CD4^+^ CD25^neg/+^ CD127^+^) from cord blood or peripheral blood from adults were co-cultured with autologous Treg cells and remaining mononuclear cells, and stimulated with *S. aureus* or *L. paracasei* for 3–4 days (as illustrated in Fig 3a). As shown in supplementary material, Fig. S1, to set a gate for CD25^+^ CD127^low^ cells (Fig. 3b) and FOXP3 expression (Fig. 3c,d) in the CellTrace violet^+^ non-Treg cell population, we compared the expression of FOXP3 or CD127 against CD25 within the total CD4^+^ T-cell population and set the gates according to the observed populations. This experimental setup revealed that *S. aureus* induced CD25^+^ CD127^low^ T cells from the CD4^+^ non-Treg cells in cell cultures from newborns, but not from adults (Fig. 3b and e). In this experiment, the vast majority of the neonatal induced CD25^+^ CD127^low^ T cells expressed FOXP3 (Fig. 3c, median value 84%) in contrast to CD25^neg/+^ CD127^+^ non-Treg cells (Fig. 3d). *Lactobacillus paracasei* did not induce FOXP3^+^ CD25^+^ CD127^low^ T cells to the same extent as stimulation with *S. aureus* and the induced fraction of these cells did not differ significantly from that in control cultures (Fig. 3b–e).

As newborn children have a higher proportion of circulating naive T cells than adults, we next examined if bacterial stimulation could convert sorted naive CD45RA^+^ non-Treg cells from adults to CD25^+^ CD127^low^ T cells. Indeed, stimulation with *S. aureus*, but not *L. paracasei*, resulted in *de novo* generation of 1·9% and 2·7% CD25^+^ CD127^low^ T cells from naive non-Treg cells in two separate experiments (Fig. 3f). However, a lower proportion of the induced CD25^+^ CD127^low^ T cells from adults expressed FOXP3 53% and 42%, respectively, in the two experiments (Fig. 3f) when compared with induced CD25^+^ CD127^low^ T cells from cord blood (median 84% Fig. 3c). With the use of CellTrace violet we could not observe any proliferation of either pre-existing or induced CD25^+^ CD127^low^ T cells in response to bacterial stimulation for 3–4 days (data not shown). Finally, there was no induction of CD25^+^ CD127^low^ T cells when CellTrace stained neonatal non-Treg cells and autologous Treg cells were stimulated with *S. aureus* in the absence of remaining mononuclear cells (see Supplementary material, Fig. S2). These results demonstrate that *S. aureus* possess an ability to convert neonatal non-Treg cells into FOXP3^+^ CD25^+^ CD127^low^ T cells via APCs.

### CD25^+^ CD127^low^ Treg cells from cultures stimulated with *S. aureus* are suppressive

As *S. aureus* converted conventional CD4^+^ T cells into FOXP3^+^ CD25^+^ CD127^low^ T cells more effectively than *L. paracasei*, we next wanted to examine whether *S. aureus*-stimulated neonatal CD25^+^ CD127^low^ T cells were suppressive. In initial experiments, cord blood mononuclear cells (*n* = 3) were cultured for 3 days in the presence or absence of *S. aureus*. From the same cord blood sample, CD4^+^ responder cells were isolated and stored at −70° during the culture period. On day 3, all CD25^+^ CD127^low^ cells in the cultures, i.e. both pre-existing and induced Treg cells, were sorted and thereafter co-cultured with autologous CD4^+^ responder cells stimulated with *α*-CD3 and *α*-CD28 (as illustrated in Fig. 4a). As shown in Fig. 4(b,c), CD25^+^ CD127^low^ T cells from unstimulated cultures suppressed the proliferation and expression of CD25 of the responder cells when cultured in a 1 : 2 and a 1 : 10 ratio. The suppressive effect of CD25^+^ CD127^low^ T cells sorted from *S. aureus*-stimulated cultures was at least as strong compared with that of Treg cells from unstimulated cultures, both at a 1 : 2 and a 1 : 10 ratio (Fig. 4b,c). Hence, these data indicate that CD25^+^ CD127^low^ Treg cells from cultures exposed to *S. aureus* keep their suppressive function.

### Stimulation of conventional CD4^+^ CD25^neg^ T cells with *S. aureus* in the absence of pre-existing tTreg cells induce activated FOXP3^low^CD25^+^ CD127^low^ T cells

As a combination of pre-existing and *S*. *aureus*-induced FOXP3^+^ CD25^+^ CD127^low^ Treg cells suppressed the proliferation of CD4^+^ responder cells, we sought to investigate the suppressive function of *S. aureus*-induced FOXP3^+^ CD25^+^ CD127^low^ T cells in the absence of pre-existing Treg cells. We were not able to perform these experiments with sorted *L. paracasei*-induced CD25^+^ CD127^low^ T cells because of the lower induction of CD25^+^ CD127^low^ T cells after stimulation with *L. paracasei* than with *S. aureus*. As illustrated in Fig. 5(a), CD4^+^ responder T cells (CD4^+^ CD25^neg^) were isolated from CBMC and stored at −70°. Thereafter, CD4^+^ CD25^+/neg^ CD127^+^ T cells (non-Treg cells), and the remaining CBMC from the same cord blood sample were sorted, co-cultured and stimulated with or without *S. aureus*. In contrast to the experiments in Fig. 3(a), the pre-existing Treg cells were not present in the cell culture. On day 3, CD25^+^ CD127^low^ T cells were sorted from *S. aureus*-stimulated cultures and co-cultured with thawed and CellTrace violet-stained CD4^+^ T cells in a ratio of 1 : 2, 1 : 4 or 1 : 5 (depending on the quantity of induced CD25^+^ CD127^low^ T cells that could be collected) in the presence of *α*-CD3 and *α*-CD28 for 4–5 days (Fig. 5a). There was no conversion of conventional CD4^+^ T cells into CD25^+^ CD127^low^ T cells in the unstimulated cell cultures (data not shown). We found that *S. aureus*-induced CD25^+^ CD127^low^ T cells from cultures without pre-existing Treg cells increased the proliferation of CD4^+^ responder cells when cultured in a ratio of 1 : 4 (Fig. 5b) and in a ratio of 1 : 2 and 1 : 5 (data not shown). Furthermore, the proportion of FOXP3^+^ cells tended to be higher in *S. aureus*-induced CD25^+^ CD127^low^ T cells from cultures with tTreg cells present during the stimulation phase compared with cultures where they were not present (Fig. 5c,d). Taken together, these results suggest that tTreg cells are important for the induction of FOXP3^+^ Treg cells in response to *S. aureus* as stimulation in the absence of tTreg cells generated an activated non-regulatory FOXP3^low^ CD25^+^ CD127^low^ T-cell phenotype.

### Blocking PD-1/PD-L1 interaction during *S. aureus* stimulation reduces the induction of neonatal FOXP3^+^ CD25^+^ CD127^low^ T cells

The interaction between PD-1 expression on activated T cells and PD-L1 expressed on APCs impedes interleukin-2 (IL-2) secretion and proliferation of CD4^+^ T cells,^34^ and might instead induce differentiation into FOXP3^+^ Treg cells.^18^ Hence, to study if the PD-1/PD-L1 axis could be involved in the induction of CD25^+^ CD127^low^ T cells, we first analysed if stimulation of mononuclear cells with *S. aureus* or *L. paracasei* could increase the expression of PD-L1 on B cells. Stimulation with *S. aureus*, but not *L. paracasei*, significantly increased the proportion of PD-L1^+^ B cells compared with controls in cultures from both newborns and adults (Fig. 6a,b). However, a significantly higher fraction of the neonatal B cells was PD-L1^+^ relative to B cells from adults (Fig. 6a,b). T cells did not up-regulate PD-L1 to the same extent as B cells upon stimulation with *S. aureus* (data not shown). PD-L1 expression was not examined on myeloid dendritic cells because of the low numbers present in the circulation. Monocytes down-regulated their specific identification markers and became highly autofluorescent in response to bacterial stimulation, which made it impossible to gate the monocyte population and subsequently the PD-L1 expression. Moreover, circulating CD25^+^ CD127^low^ Treg cells expressed PD-1 to a greater extent than CD4^+^ non-Treg cells in newborns, but not in adults (Fig. 6c). After bacterial stimulation, the proportion of PD-1^+^ cells was higher in the induced CD25^+^ CD127^low^ T cells compared with conventional CD4^+^ T cells (Fig. 6d,e). Finally, the induction of CD25^+^ CD127^low^ T cells was reduced or completely inhibited when blocking PD-L1 with anti-PD-L1, but not with isotype antibody control (Fig. 6f,g). Taken together, these results demonstrate that the PD-1/PD-L1 axis is involved in the induction of FOXP3^+^ CD25^+^ CD127^low^ T cells after *S. aureus* stimulation.

## Discussion

In the present study we investigated whether commensal gut bacteria could induce FOXP3^+^ Treg cells. We here demonstrate that stimulation with *S. aureus* converts neonatal CD4^+^ CD25^neg^ T cells into FOXP3^+^ CD25^+^ CD127^low^ T cells. We also show that APCs expressing PD-L1 were pivotal for this induction as blocking the interaction between PD-1 and PD-L1 reduced the proportion of FOXP3^+^ CD25^+^ CD127^low^ T cells.

Until recently, tTreg cells were identified by a series of naive T-cell markers, such as CD45RA, CD62L and CCR7, as well as the recent thymic emigrant molecule CD31. However, it has been suggested that Helios might serve as a marker to distinguish thymically derived from peripherally induced Treg cells.^11^ Here we show for the first time that a significantly higher proportion of the neonatal Treg cells expressed Helios relative to those in adults. However, Helios can also be induced following activation in human CD4^+^ T cells.^12^ Still, over 80% of the CD4^+^ T cells in cord blood are recent thymic emigrants that express CD31^35^ and we found that the majority of the neonatal Treg cells were of a naive CD45RO-negative phenotype. Therefore, we consider the Helios^+^ CD25^+^ CD127^low^ T-cell population found in newborns to be tTreg cells, rather than newly activated T cells, and that there is a higher proportion of these cells in the circulation early compared with later in life.

Mice raised under sterile conditions have a lower proportion of functional FOXP3^+^ Treg cells in the gut compared with wild-type mice.^25^ Colonization of the gut with either a mixture of different bacterial strains, a mixture of *Clostridium* species or with *Bacteroides fragilis* stimulates the induction of FOXP3^+^ Treg cells in the intestine of these mice.^23^–^26^ In infants, early intestinal colonization of the gut with *S. aureus* has been shown to be negatively associated with food allergy development in children.^29^,^36^ Also, administration of lactobacilli (*L. reuteri*) was negatively associated with IgE-mediated eczema.^30^ Therefore, we hypothesized that stimulation with these two gut commensals would induce Treg cells in newborn infants. However, as it is impossible to mono-colonize the intestine of human infants *in vivo* with particular bacterial strains, we resolved to study the effects of bacterial stimulation on neonatal CD4^+^ T cells *in vitro*.

Our initial experiments showed that conventional CD4^+^ T cells from cord blood are more prone to undergo apoptosis compared with Treg cells after primary *in vitro* stimulation with different antigens, as also shown by others.^37^ Indeed, analysing the proportion of Treg cells after bacterial stimulation of mononuclear cells resulted in a false increase of these cells in stimulated cultures compared with control cultures (our unpublished data). We therefore used an experimental model in which the conventional CD4^+^ T cells could be traced allowing us to distinguish between induced and pre-existing tTreg cells. For the first time we here show that stimulation with *S. aureus* converts neonatal conventional CD4^+^ T cells into FOXP3^+^ CD25^+^ CD127^low^ T cells. In cell cultures from adults, *S. aureus* stimulation was able to induce CD25^+^ CD127^low^ T cells only if sorted naive T cells were used, but adult T cells expressed FOXP3 to a lesser extent than those from cord blood. These results suggest that it is mainly the naive T-cell population that differentiates into CD25^+^ CD127^low^ T cells upon stimulation with *S. aureus* and that the induction of FOXP3-expressing putative Treg cells occurs primarily after stimulation of neonatal T cells. Regarding stimulation with *L. paracasei*, others have shown that *Lactobacillus acidophilus* is a potent Treg cell inducer in mononuclear cell cultures from adults, but that different lactobacillus strains differ in their ability to induce Treg cells.^31^ This might explain why we only observed a statistically non-significant tendency for induction of Treg cells after stimulation with *L. paracasei*.

Antigen-presenting cells are activated by *S. aureus* in an innate fashion via pattern recognition receptors, including TLR2.^38^ Moreover, staphylococcal protein A, which is expressed by > 90% of human *S. aureus* isolates,^39^ is referred to as a B-cell superantigen.^40^ Activated APCs secrete pro-inflammatory cytokines and up-regulate MHC and co-stimulatory molecules on the cell surface. The cytokine milieu in response to different bacteria is crucial for the accurate differentiation of effector T cells, but certain cytokines have also been found to inhibit the induction and reduce the function of Treg cells. During inflammation the regulatory function of Treg cells is reduced^41^ and the inflammatory cytokine tumour necrosis factor (TNF) has been found to inhibit the suppressive function of both tTreg cells and induced Treg cells via the TNFRII that is constitutively expressed on naive Treg cells.^42^ Moreover, IL-6 can impede conversion of conventional CD4^+^ T cells to FOXP3^+^ Treg cells.^43^ *Staphylococcus aureus*, as well as other Gram-positive bacteria, induce secretion of both TNF and IL-6 by mononuclear cells from adults,^44^ which we also detect in cell cultures from newborn children (our unpublished data). In spite of this, *S. aureus* stimulation did not hamper the regulatory function of pre-existing Treg cells nor prohibit the conversion of neonatal conventional CD4^+^ T cells to FOXP3^+^ CD25^+^ CD127^low^ T cells.

High expression of FOXP3 in CD4^+^ CD25^+^ T cells has been found to be connected with the suppressive capacity of Treg cells, whereas low FOXP3 expression is associated with an activated T-cell phenotype.^45^ We observed that *S. aureus* converted conventional CD4^+^ T cells to an activated non-suppressive FOXP3^low^ CD25^+^ CD127^low^ phenotype if tTreg cells were depleted during the stimulation phase, which indicates that pre-existing Treg cells are needed to induce functional Treg cells. There are several mechanisms by which tTreg cells could influence the cell culture environment and so contribute to the Treg cell induction. Thymically derived Treg cells that are cultured with autologous CD4^+^ CD25^neg^ T cells convey suppressive function to the conventional T cells that starts to secrete the regulatory cytokines IL-10^46^ or transforming growth factor-*β*,^47^ a phenomenon referred to as infectious tolerance. Furthermore, Treg cells co-cultured with dendritic cells will down-regulate the maturation markers HLA-DR, CD83 and CD80 of the dendritic cells, resulting in tolerogenic dendritic cells that secrete IL-10.^48^ Therefore, it is possible that the presence of tTreg cells is required to generate a regulatory cytokine milieu that facilitates conversion of CD4^+^ T cells into functional FOXP3^+^ Treg cells during *S. aureus* stimulation. To formally confirm this theory it would have been necessary to study the suppressive function of *S. aureus*-induced CD25^+^ CD127^low^ T cells with tTreg cells present during the stimulation phase but not during the suppression experiments. This experiment is exceedingly difficult to perform with cells from human infants because it requires a large amount of mononuclear cells from cord blood that can barely be obtained.

A major role of the PD-1 and PD-L1 engagement is to limit T-cell activation^34^,^49^ and induce FOXP3^+^ Treg cells.^18^–^20^ The molecular mechanism behind this induction has been suggested to involve recruitment of phosphatases within the T cells that will inhibit the T-cell receptor signalling pathway.^18^,^19^,^21^ Interestingly, we found that *S. aureus* considerably increased the proportion of PD-L1^+^ B cells, but to a greater degree in cultures from newborns than in those from adults, and that PD-1 was up-regulated in induced neonatal CD25^+^ CD127^low^ T cells compared with non-Treg cells after bacterial stimulation. In line with this, *S. aureus* stimulation of monocytes from adults up-regulates PD-L1, but down-regulates the activation marker CD80 and MHC-II molecules on the cell surface.^38^ Moreover, blocking PD-L1 decreased or completely inhibited the induction of CD25^+^ CD127^low^ T cells after stimulation with *S. aureus*, which points to the possibility that the PD-1/PD-L1 axis could be one of the mechanisms by which APCs induce FOXP3^+^ CD25^+^ CD127^low^ T cells after stimulation with *S. aureus*. Hence, these findings further argue for the *de novo*-generated FOXP3^+^ CD25^+^ CD127^low^ T cells in response to *S. aureus* stimulation being Treg cells rather than activated T cells.

In conclusion, this study demonstrates that Helios might be used as a marker to identify tTreg cells in healthy newborn children and that newborns have a higher proportion of tTreg cells in the circulation compared with adults. We also show that the commensal gut bacteria *S. aureus* convert conventional neonatal CD4^+^ T cells to FOXP3^+^ CD25^+^ CD127^low^ T cells, which was dependent on the presence of both tTreg cells and of PD-L1-expressing APCs. We suggest that *S. aureus* might directly influence both Treg cells and APCs and that the interplay between these cell populations results in the induction of Treg cells.

## Abbreviations

APC: antigen-presenting cell
CBMC: cord blood mononuclear cell
CTLA-4: cytotoxic T-lymphocyte antigen 4
DC: dendritic cell
FOXP3: forkhead box P3
IL-2: interleukin-2
PBMC: peripheral blood mononuclear cell
PD-1: programmed cell death 1
PD-L1: programmed cell death ligand 1
PE: phycoerythrin
pTreg: peripherally derived regulatory T cell
TNF: tumour necrosis factor
tTreg: thymically derived regulatory T cell

## Supporting Information

**Figure S1.** Gating strategy for CD25^+^ CD127^low^ T cells and FOXP3^+^ CD25^+^ T cells. **Figure S2.** *Staphylococcus aureus* stimulation of preexisting regulatory T (Treg) cells and non-Treg cells in the absence of antigen-presenting cells.
