# Supplementary material for: Staphylococcus aureus convert neonatal conventional CD4+ T cells into FOXP3+ CD25+ CD127low T cells via the PD-1/PD-L1 axis
Source: Immunology. 2014 Feb 10;141(3):467–81. doi: 10.1111/imm.12209 (PMC3930383; doi:10.1111/imm.12209)
Supplement: Supplementary file 1 — Figure S1. Gating strategy for CD25+ CD127low T cells and FOXP3+ CD25+ T cells. Figure S2. Staphylococcus aureus stimulation of preexisting regulatory T (Treg) cells and non-Treg cells in the absence of antigen-presenting cells. [file imm0141-0467-sd1.ppt]

## Slide 1
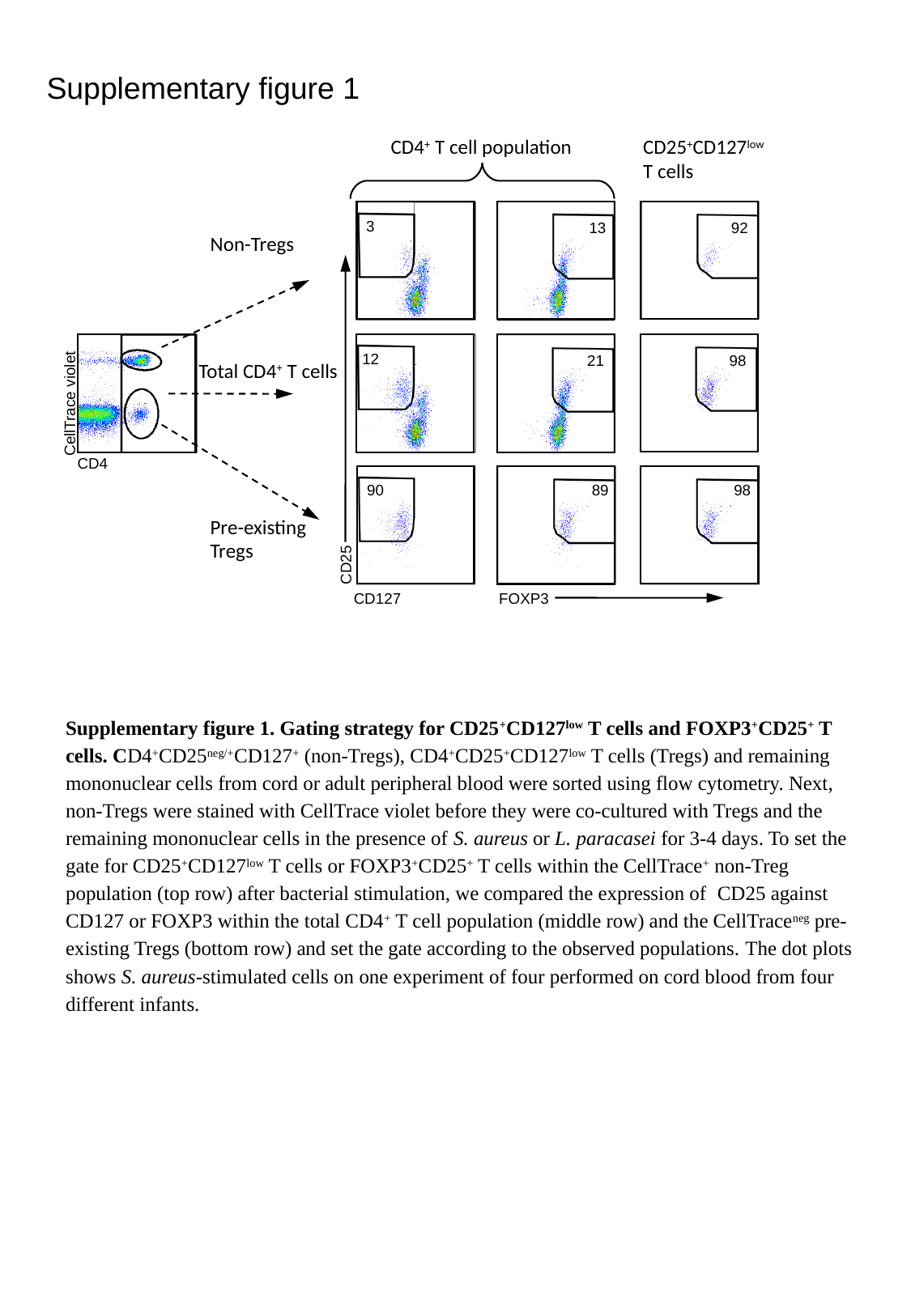

Supplementary figure 1
CD4+ T cell population
CD25+CD127low
T cells
3
13
92
Non-Tregs
12
21
98
Total CD4+ T cells
CellTrace violet
CD4
98
89
90
CD25
Pre-existing
Tregs
CD127
FOXP3
Supplementary figure 1. Gating strategy for CD25+CD127low T cells and FOXP3+CD25+ T cells. CD4+CD25neg/+CD127+ (non-Tregs), CD4+CD25+CD127low T cells (Tregs) and remaining mononuclear cells from cord or adult peripheral blood were sorted using flow cytometry. Next, non-Tregs were stained with CellTrace violet before they were co-cultured with Tregs and the remaining mononuclear cells in the presence of S. aureus or L. paracasei for 3-4 days. To set the gate for CD25+CD127low T cells or FOXP3+CD25+ T cells within the CellTrace+ non-Treg population (top row) after bacterial stimulation, we compared the expression of CD25 against CD127 or FOXP3 within the total CD4+ T cell population (middle row) and the CellTraceneg pre-existing Tregs (bottom row) and set the gate according to the observed populations. The dot plots shows S. aureus-stimulated cells on one experiment of four performed on cord blood from four different infants.

## Slide 2
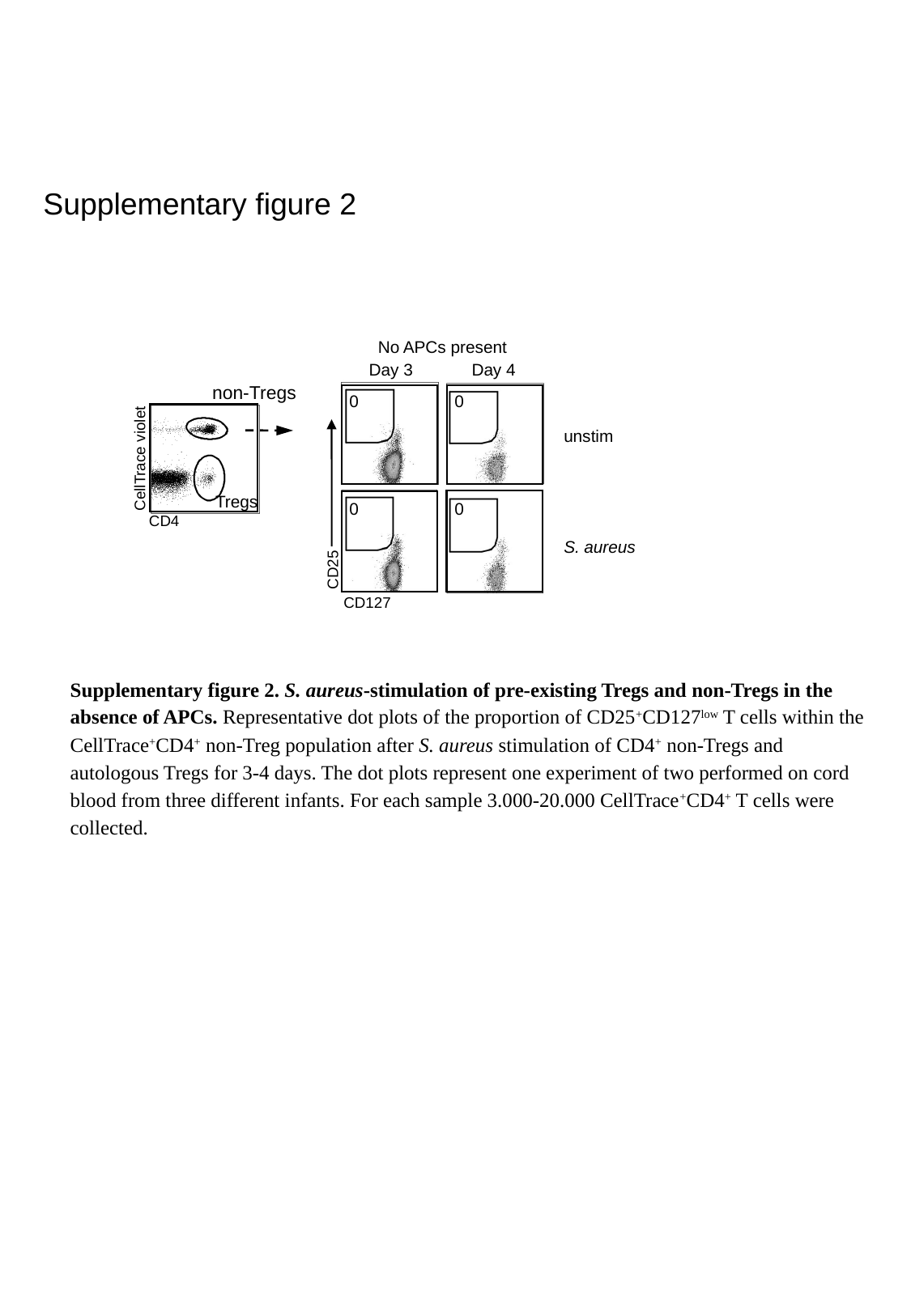

Supplementary figure 2
No APCs present
Day 3
Day 4
0
0
0
0
unstim
S. aureus
CD25
CD127
non-Tregs
CellTrace violet
Tregs
CD4
Supplementary figure 2. S. aureus-stimulation of pre-existing Tregs and non-Tregs in the absence of APCs. Representative dot plots of the proportion of CD25+CD127low T cells within the CellTrace+CD4+ non-Treg population after S. aureus stimulation of CD4+ non-Tregs and autologous Tregs for 3-4 days. The dot plots represent one experiment of two performed on cord blood from three different infants. For each sample 3.000-20.000 CellTrace+CD4+ T cells were collected.
